# Oxygen Saturation on Admission Is a Predictive Biomarker for PD-L1 Expression on Circulating Monocytes and Impaired Immune Response in Patients With Sepsis

**DOI:** 10.3389/fimmu.2018.02008

**Published:** 2018-09-04

**Authors:** José Avendaño-Ortiz, Charbel Maroun-Eid, Alejandro Martín-Quirós, Roberto Lozano-Rodríguez, Emilio Llanos-González, Víctor Toledano, Paloma Gómez-Campelo, Karla Montalbán-Hernández, César Carballo-Cardona, Luis A. Aguirre, Eduardo López-Collazo

**Affiliations:** ^1^Innate Immunity Group, IdiPAZ, La Paz University Hospital, Madrid, Spain; ^2^Tumor Immunology Lab, IdiPAZ, La Paz University Hospital, Madrid, Spain; ^3^Center for Biomedical Research Network, CIBERES, Madrid, Spain; ^4^Emergency Department, IdiPAZ, La Paz University Hospital, Madrid, Spain

**Keywords:** monocytes, sepsis, PD-L1, oxygen saturation, T cell exhaustion, hypoxemia

## Abstract

Sepsis is a pathology in which patients suffer from a proinflammatory response and a dysregulated immune response, including T cell exhaustion. A number of therapeutic strategies to treat human sepsis, which are different from antimicrobial and fluid resuscitation treatments, have failed in clinical trials, and solid biomarkers for sepsis are still lacking. Herein, we classified 85 patients with sepsis into two groups according to their blood oxygen saturation (SaO_2_): group I (SaO_2_ ≤ 92%, *n* = 42) and group II (SaO_2_ > 92%, *n* = 43). Blood samples were taken before any treatment, and the immune response after *ex vivo* LPS challenge was analyzed, as well as basal expression of PD-L1 on monocytes and levels of sPD-L1 in sera. The patients were followed up for 1 month. Taking into account reinfection and *exitus* frequency, a significantly poorer evolution was observed in patients from group I. The analysis of HLA-DR expression on monocytes, T cell proliferation and cytokine profile after *ex vivo* LPS stimulation confirmed an impaired immune response in group I. In addition, these patients showed both, high levels of PD-L1 on monocytes and sPD-L1 in serum, resulting in a down-regulation of the adaptive response. A blocking assay using an anti-PD-1 antibody reverted the impaired response. Our data indicated that SaO_2_ levels on admission have emerged as a potential signature for immune status, including PD-L1 expression. An anti-PD-1 therapy could restore the T cell response in hypoxemic sepsis patients with SaO_2_ ≤ 92% and high PD-L1 levels.

## Background

Sepsis is life-threatening organ dysfunction caused by a dysregulated host response to infection (1); it is currently a leading cause of death in intensive care units worldwide (2). Several therapeutic strategies to treat human sepsis, which are different from antimicrobial and fluid resuscitation treatments, have failed in clinical trials, and solid biomarkers for sepsis are still lacking (3).

Two phases have been recognized in this disease: an early inflammatory phase and a late immunosuppressive stage (4–6); however, these two phases can overlap (4, 7). In this regard, monocytes/macrophages are believed to play an important role in orchestrating the host immune response during sepsis (4, 7). They participate in both phases of sepsis by firstly releasing inflammatory cytokines that contribute to a *cytokine storm*, and secondly adopting an immune depressive phenotype, whereupon they are unable to respond to secondary infections (4). Decreased human leukocyte antigen (HLA)-DR expression has also been reported during the immunosuppressive phase in monocytes, reducing the switch to the adaptive response (4). In addition, polarization of the adaptive response has been reported in lipopolysaccharide (LPS)-injected healthy donors and in murine polymicrobial sepsis (8). These observations highlight the importance of the interaction between monocytes and lymphocytes and its role in T cell exhaustion (6). The discovery and characterisation of immune checkpoints (ICs) adds a new parallel window of study in which cell-to-cell interaction could have an important role beyond cancer pathologies (9). In this regard, we and others have already reported programmed death-ligand 1 (PD-L1) overexpression on sepsis monocytes (10–13), which was associated with risk stratification and mortality in these patients (14).

In accordance with our previous data, the biological activities described above are controlled by hypoxia-inducible factor-1α (HIF1α) expression (13, 15). In this regard, HIF1α is the most important pathway for oxygen homeostasis in mammals (16). Under normoxia, oxygen and prolyl hydroxylases (PHDs) hydroxylate the HIF1α subunit inducing its ubiquitination (17). Under hypoxic conditions, this hydroxylation is inhibited and HIF1α accumulates and translocates to the nucleus activating a number of significant pathways (16, 17). In blood, an abnormal low level of oxygen is known as hypoxemia. Although traditionally hypoxemia was defined as an oxygen saturation (SaO_2_) <90%, a hospital admission threshold of 92% showed be safer (18). How hypoxia and HIF1α affect the course of infections remains unclear (19). Therefore, although hypoxemia is considered a bad prognostic marker in sepsis, a clear association to immune status and outcome in sepsis patients is still lacking.

Herein, in order to study the association of hypoxemia with immune alterations, we classified 85 patients with sepsis according to their oxygen saturation (SaO_2_) on admission and analyzed their immunological response. Additionally, *ex vivo* PHDs inhibition using Dimethyloxaloylglycine (DMOG) were used to stablish causal relationship between both features. Our data open a new window of immunotherapy treatment for patients with sepsis, based on their SaO_2_ level upon admission.

## Methods

### Study design

Eighty-five patients who fulfilled the diagnostic criteria for sepsis according to the Society of Critical Care Medicine and the European Society of Intensive Care Medicine international conferences (20, 21) were included in the study. Blood samples were collected at the time of admission, before any therapy, and sepsis was confirmed using clinical and analytical data. Exclusion criteria: chronic inflammatory diseases (except asthma), presence of hematological malignancies, treatment with steroids and/or immunosuppressive drugs in the last month, previous presence of severe liver failure (serum aspartate aminotransferase and/or alanine aminotransferase >100 IU/L, prothrombin time <60% and total bilirubin <60 mmol/L), renal failure (plasma creatinine >200 μmol/L), HIV/AIDS, hepatitis B or C and pregnancy. On admission and previously to any treatment the SaO_2_ of the 85 patients were measured by pulse-oximetry, then sepsis being classified into two groups according to their SaO_2_. The clinical data of the patients included in the study are summarized in Table 1. Patients were followed up for 1 month and any reinfection events and *exitus* were reported. Blood samples from healthy volunteers (HV, *n* = 15) that matched with patients in age, sex and body mass index were collected from the blood donor service of La Paz University Hospital.

**Table 1:** Patient characteristics.

	**Group I SaO_2_ ≤ 92% (*n* = 42)**	**Group II SaO_2_ > 92% (*n* = 43)**		**Logistic Regression**	
				**OR (95% CI)**	***p*-value**
O_2_ saturation, %	85.24 ± 7.12	96.38 ± 1.91			
Age, years	76.06 ± 15.67	62.4 ± 22.25			
Sex, male, *n* (%)	24 (57.2)	17 (39.5)			
**Comorbidities**, ***n*** **(%)**			***p*****-value**		
Hypertension	35 (83)	24 (55.8)	0.240		
Diabetes mellitus	20 (47)	7 (16.3)	0.008^*^		
Current smoking	3 (7.1)	6 (13.9)	0.223		
Current alcoholism	3 (7.1)	2 (4.7)	0.735		
Chronic kidney disease	7 (16.7)	6 (13.9)	0.925		
CVD	18 (42.9)	11 (25.5)	0.222		
COPD	12 (28.5)	5 (11.6)	0.102		
**APACHE II**	20.92 ± 6.67	13.33 ± 5.24	<0.001^*^		
**q-SOFA**, ***n*** **(%)**			<0.001^*^		
0	1 (2.4)	4 (9.3)			
1	6 (14.3)	21 (48.8)			
2	28 (66.7)	18 (41.9)			
3	15 (35.7)	2 (4.7)			
Glasgow	12.5 ± 2.46	14.4 ± 1	<0.001^*^		
Temperature, °C	37.97 ± 1.53	37.57 ± 1.54	0.210		
Glucose, mg/dL	176.36 ± 145.55	139.27 ± 61.44	0.104		
MBP, mm Hg	70.24 ± 19.26	64.87 ± 13.25	0.121		
SBP, mm Hg	105.7 ± 30.16	94.1 ± 18.87	0.026^*^		
Heart rate, bpm	102.64 ± 22.73	102.22 ± 25.13	0.932		
Respiratory rate, brpm	28.12 ± 5.11	21.87 ± 4.25	<0.001^*^	669 (0.579, 0.837)	**0.000**
Hemoglobin, units	12.26 ± 2.68	13.33 ± 2.24	0.039^*^		
Hematocrit, %	37.91 ± 9.11	40.40 ± 6.2	0.127		
Lactate, nmol/L	3.54 ± 3.88	3.48 ± 2.63	0.930		
LDH, UI/L	146.79 ± 188.81	218.62 ± 205.25	0.215		
Serum creatinine, mg/dL	2.05 ± 1.51	1.49 ± 0.76	0.022^*^	0.487 (0.265, 0.897)	**0.021**
CRP, mg/L	171.34 ± 113.88	162.77 ± 114.62	0.128		
GOT	205.96 ± 648.01	83.91 ± 173.04	0.209		
GTP	128.1 ± 337.22	95.38 ± 239.97	0.592		
Bilirubin	2.11 ± 7.05	1.88 ± 2.47	0.829		
HCO_3_, mEq/L	22.29 ± 6.98	22.64 ± 3.97	0.758		
Na, mEq/L	139.48 ± 9.02	134.68 ± 3.7	0.001^*^	0.802 (0.661, 0.973)	**0.026**
K, mEq/L	4.3 ± 1.02	3.9 ± 0.58	0.020^*^		
pH	7.38 ± 0.15	7.39 ± 0.07	0.610		
INR	1.626 ± 1.23	1.44 ± 1.14	0.450		
Length of stay, days	9.92 ± 8	12.56 ± 13.747	0.256		
**Immune System**					
sPDL1	8.32 ± 3.20	5.08 ± 2.36	0.0033^*^	0.622 (0.461, 0.839)	**0.002**
mPDL1 (%)	15.84 ± 11.69	6.88 ± 6.77	0.0005^*^		
**Type of sepsis**			0.470		
Severe sepsis	28 (66.7%)	30 (69.8%)			
Septic shock	14 (33.3%)	13 (30.2%)			

*Data are presented as mean ± SD, or number (%). T-test with previous Levene test or chi-squared test of ≤ 92% vs. >92% subgroups where appropriate. Statistically significant p-values (p < 0.05) using Student's t-test and logistic regression model analysis are in bold. OR, odds ratio; CVD, cardiovascular disease; COPD, chronic obstructive pulmonary disease; APACHE II, Acute Physiology and Chronic Health Evaluation II; qSOFA, quick Sequential Organ Failure Assessment; MBP, mean blood pressure; SBP, systolic blood pressure; bpm, beats per minute; brpm, breaths per minute; LDH, lactate dehydrogenase; CRP, C-reactive protein; INR, international normalized ratio*.

The Committee for Human Subjects of La Paz University Hospital approved the study, which was conducted in accordance with the ethical guidelines of the 1975 Declaration of Helsinki. The participants provided written consent for the study.

### Reagents

Roswell Park Memorial Institute (RPMI) medium (Invitrogen) was used for the cell cultures. The following antibodies were used: anti-CD14, anti-HLA-DR, anti-CD3 (Immunostep), and anti-PD-L1 (Miltenyi Biotec). The LPS from *Salmonella abortus* was a kind gift from Dr. Galanos (Max Planck Institute of Immunobiology and Epigenetics). Carboxyfluorescein succinimidyl ester (CFSE) for the proliferation assays was purchased from Thermo Fisher. The lymphocyte stimulus pokeweed (PWD) was purchased from Sigma-Aldrich. To inhibit PD-1/PD-L1 interaction, a fully human IgG4 (S228P) anti-PD-1 receptor-blocking monoclonal antibody was used (Bristol-Myers Squibb). All the reagents used for cell cultures were endotoxin-free, as assayed with the *Limulus* amebocyte lysate test (Cambrex).

### Monocyte and lymphocyte isolation from peripheral blood

The peripheral blood mononuclear cells (PBMCs) were isolated using Ficoll-Plus gradient (GE Healthcare Bio-Sciences) as reported previously (4, 13).

### Cytometric bead array

Tumor necrosis factor alpha (TNFα), interleukins (IL)1β, IL6, and IL10 protein levels in the culture supernatants were determined using the Human Inflammatory cytometric bead array (CBA) kit (BD Biosciences).

### Flow cytometry analysis

For markers staining, the cells were labeled with: allophycocyanin (APC)-conjugated anti-human CD14, fluorescein isothiocyanate (FITC)-conjugated anti-human HLA-DR, APC-conjugated anti-human CD3 (all from Immunostep, Spain); and phycoerythrin (PE)-conjugated anti-human PD-L1 (Miltenyi Biotec, USA). Matched isotype antibodies were used as negative controls. The cells were incubated for 30 min at 4°C in the dark. The data were acquired by flow cytometry using a BD FACSCalibur flow cytometer (BD Biosciences) and analyzed with FlowJo vX.0.7 software (FlowJo, LLC). Gating strategy is shown in Supplementary Figure 1.

### Soluble PD-L1 measurement

Soluble PD-L1 (sPD-L1) on patients with sepsis and HV sera was measured using an enzyme-linked immunosorbent assay (PDL1 ELISA Kit, Cloud-Clone Corp., USA).

### T cell proliferation assays

Proliferation was analyzed by flow cytometry of CFSE-labeled cells, as reported previously (13). Briefly, PBMCs from patients were labeled with CFSE and 10^5^ PBMCs per well were seeded in a round bottom p96 plate (Corning costar, USA) and stimulated with 2.5 μg/mL of PWD and treated or not with 5 μg/ml of fully human IgG4 (S228P) anti-PD-1 monoclonal antibody (Bristol-Myers Squibb) during 5 days.

### DMOG *in vitro* model

Monocytes were isolated from HV peripheral blood by centrifugation on Ficoll-Hypaque Plus (Amersham Biosciences) and adherence, as we have described before (13).The composition of this adherent population of cells was analyzed by FACS. Once seeded, adherent cells were treated with 100 μM of DMOG two hours before 24 h of 10 ng/mL LPS stimulation. Cytokine productions on supernatant and cell surface markers were checked by CBA and cytometric analysis.

### RNA isolation and quantification

The cells were washed once with PBS and the RNA was isolated using the High Pure RNA Isolation Kit (Roche Diagnostics). The real-time quantitative PCRs were performed using the QuantiMix Easy SYG kit from Biotools and specific primers as described previously (4, 15, 22–25).

### Statistical analysis

The number of patients analyzed is indicated in each figure. Descriptive statistics are presented as counts and percentages, or as means with standard deviation as appropriated. Comparisons of subgroups were made using unpaired Student's *t*-test for quantitative variables or χ^2^ test for categorical variables. We studied univariate associations between sPD-L1 and PD-L1 and oxygen saturation, using the Pearson's correlation coefficients. Moreover, we selected membrane PD-L1, sPD-L1, and clinical parameters showing significant differences between normoxemic and hypoxemic groups and fitted a logistic regression model. The severity scores APACHE II was not taking into account in this analysis because it includes SaO_2_. The statistical significance was set at *p* < 0.05. The analyses were conducted using Prism 5.0 software (GraphPad) and SPSS version 23 (IBM) software.

## Results

### Low oxygen saturation is associated to poor prognosis and higher rate of secondary infections in patients with sepsis

**Figure 1:**
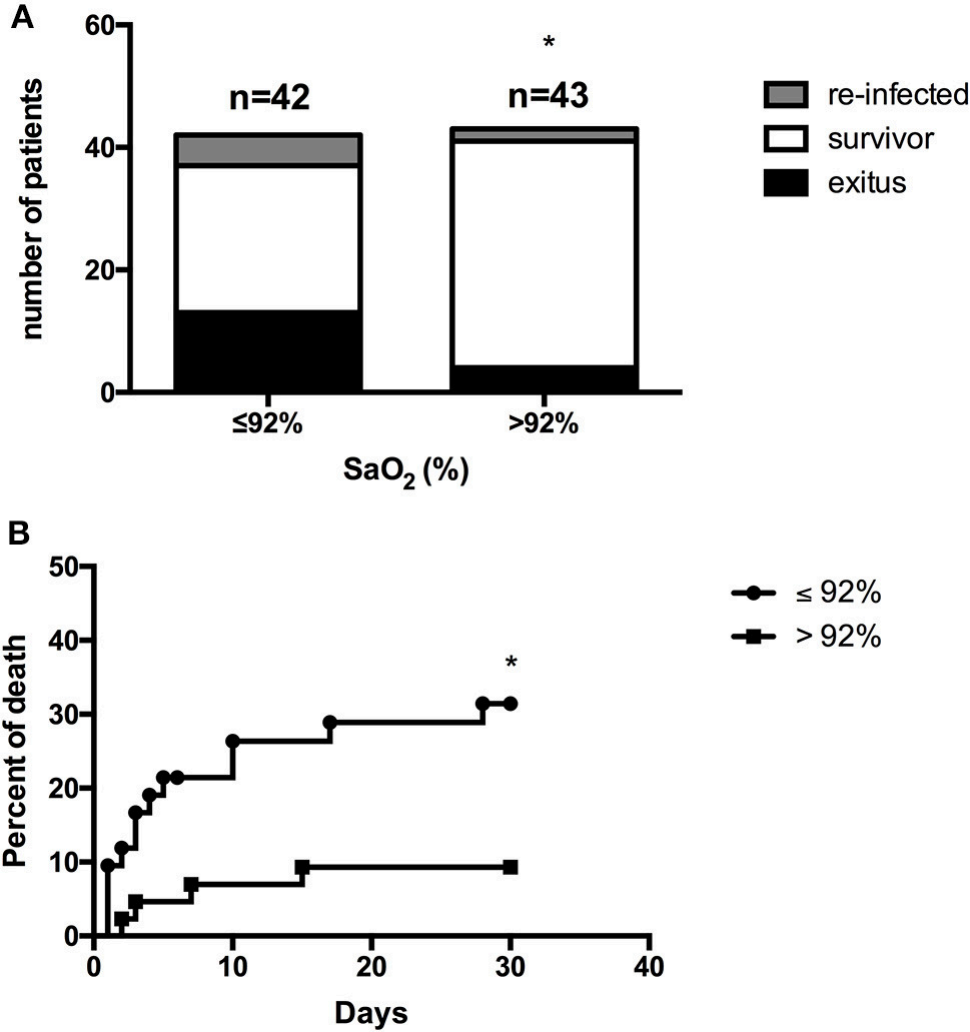
Frequency of survivors, survivors re-infected and *exitus* in patients with sepsis classified according to their oxygen saturation.Patients with sepsis (*n* = 85) were classified into two groups according to their oxygen saturation (SaO_2_) on admission (≤92% and >92%), and were followed up for 1 month. **(A)** The number of survivors, survivors who had at least one reinfection episode and *exitus* are reported. ^*^χ^2^ = 13.078; *P* = 0.001. **(B)** The percentage of death accumulated is shown. ^*^χ^2^ = 5.708; *P* = 0.017.

Eighty-five patients with sepsis were classified according to their SaO_2_ into two groups (group I, SaO_2_ ≤ 92% and group II, SaO_2_ > 92%). Patients in group I exhibited a number of clinical parameters that match with a poor prognosis (e.g., APACHEII, q-SOFA and Glasgow score, Table 1). This result was reflected in the patient evolution, as evidenced by the number of survivors who suffered reinfection and *exitus* in both groups (Figure 1A). In this regard, the statistical analysis also indicated that there was a significant difference between groups I and II (χ^2^ = 13.078; *P* = 0.001). Similarly, the percentage of death was significantly higher in group I than group II, (χ^2^ = 5.708, *P* = 0.017) (Figure 1B).

### Low oxygen saturation is associated to impaired immune response in septic patients

**Figure 2:**
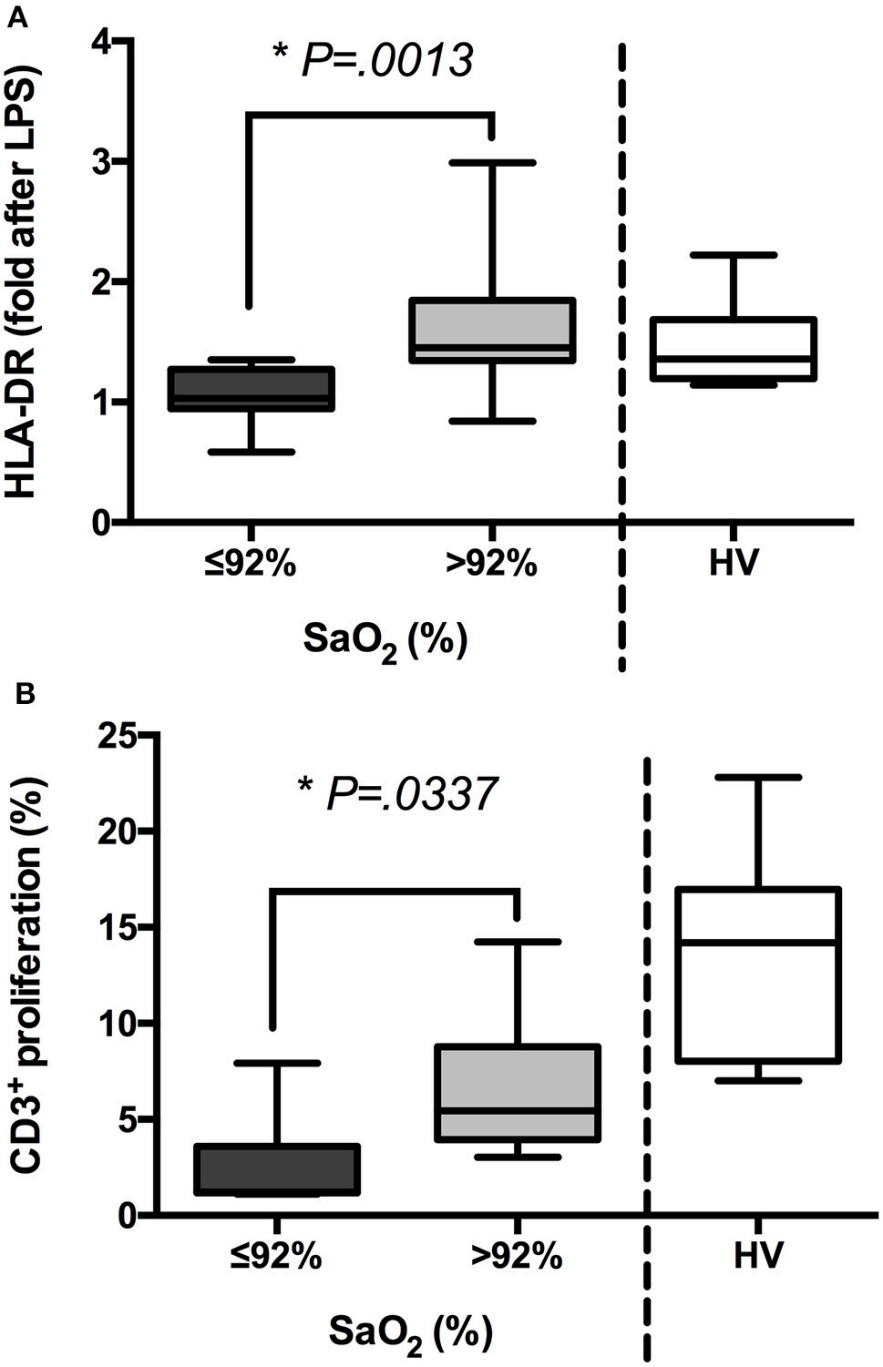
The groups of patients with sepsis exhibit different states of activation after *ex vivo* challenge. **(A)** Blood samples from patients with sepsis (*n* = 85) and healthy volunteers (HV, *n* = 15) were stimulated or not with LPS (5 ng/mL, 3 h) *ex vivo*. Then, mean intensity of fluorescence (MIF) of HLA-DR on the gate of CD14^+^ cells was analyzed by FACS. Folds after LPS challenge are shown in patients classified according to their oxygen saturation and HV. **(B)** PBMCs were isolated from patients with sepsis (*n* = 85) and HV (*n* = 15), labeled with CFSE and stimulated or not with PWD (2.5 μg/mL) for 5 days. Then, proliferation of CD3^+^ cells was analyzed by FACS. Percentages of proliferation are shown in patients classified according to their oxygen saturation and HV ^*^p < 0.05 using a Student's *t*-test.

**Figure 3:**
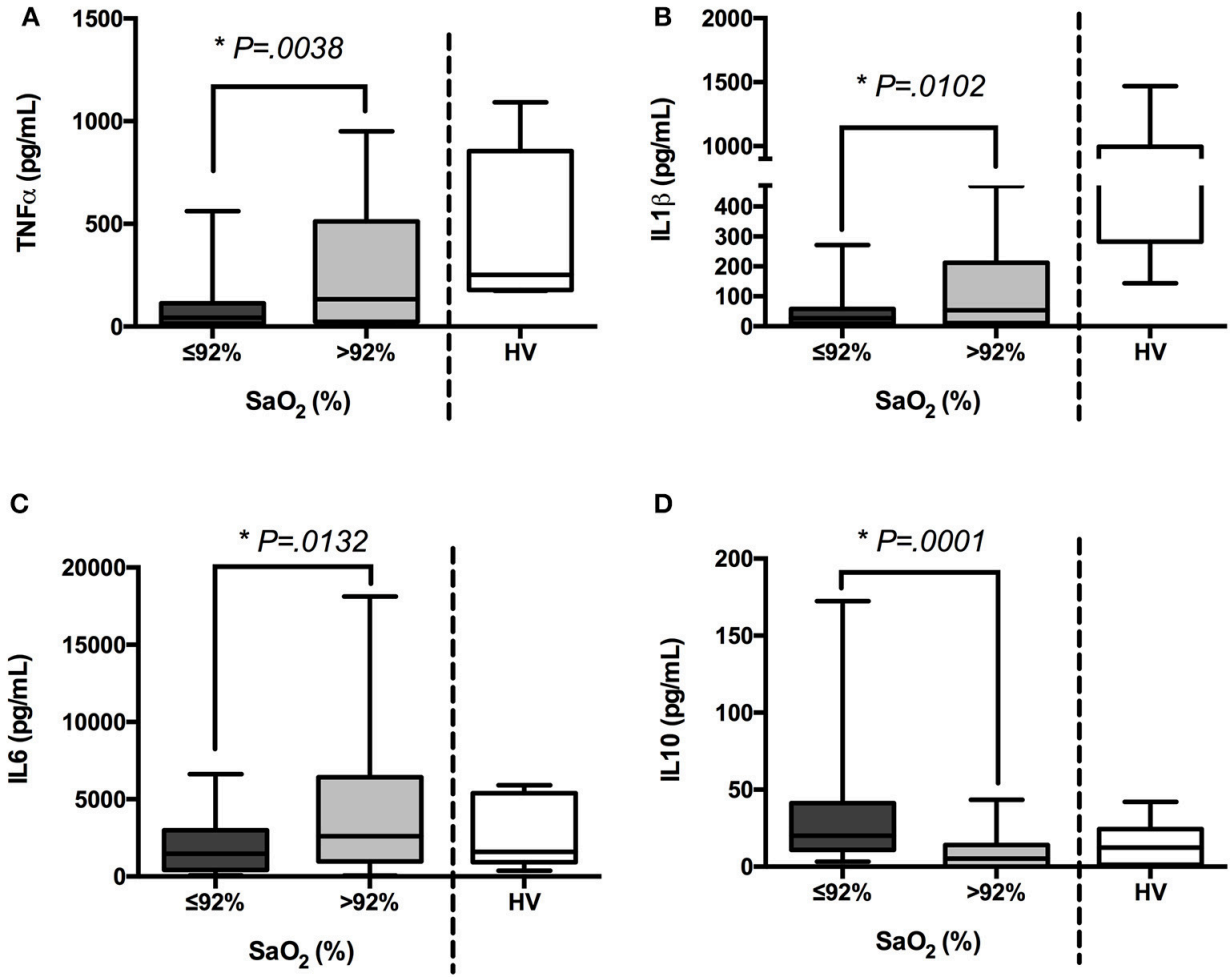
Inflammatory response is donwregulated in patients with sepsis who exhibited low oxygen saturation. TNFα **(A)**, IL-1β **(B)**, IL-6 **(C)**, and IL10 **(D)** production (pg/mL) after 3 h (TNFα, IL-6 and IL10) or 6 h (IL1β) of *ex vivo* LPS stimulation (5 ng/mL) in whole blood samples from patients with sepsis (*n* = 85) and healthy volunteers (HV, *n* = 15), were quantified by cytometric bead array (CBA) and FACS. Cytokine quantifications in patients classified according to their oxygen saturation and HV are shown. ^*^p < 0.05 using a Student's *t*-test.

Patients under 92% SaO_2_ (group I) showed a patent impaired immune response. Their antigen presentation was affected due to reduced HLA-DR expression on CD14^+^ cells after *ex vivo* LPS challenge (Figure 2A). Thus, when their CFSE-labeled PBMCs were stimulated with PWD, it resulted in a significant decrease in T cell proliferation (Figure 2B). Moreover, in a whole blood assay, LPS stimulation did not upregulate proinflammatory cytokine levels (TNFα, IL1β, and IL6) but IL10 ones in samples from this group (Figure 3).

### PD-L1 expression on monocytes and sPD-L1 levels in sera inversely correlate to oxygen saturation

**Figure 4:**
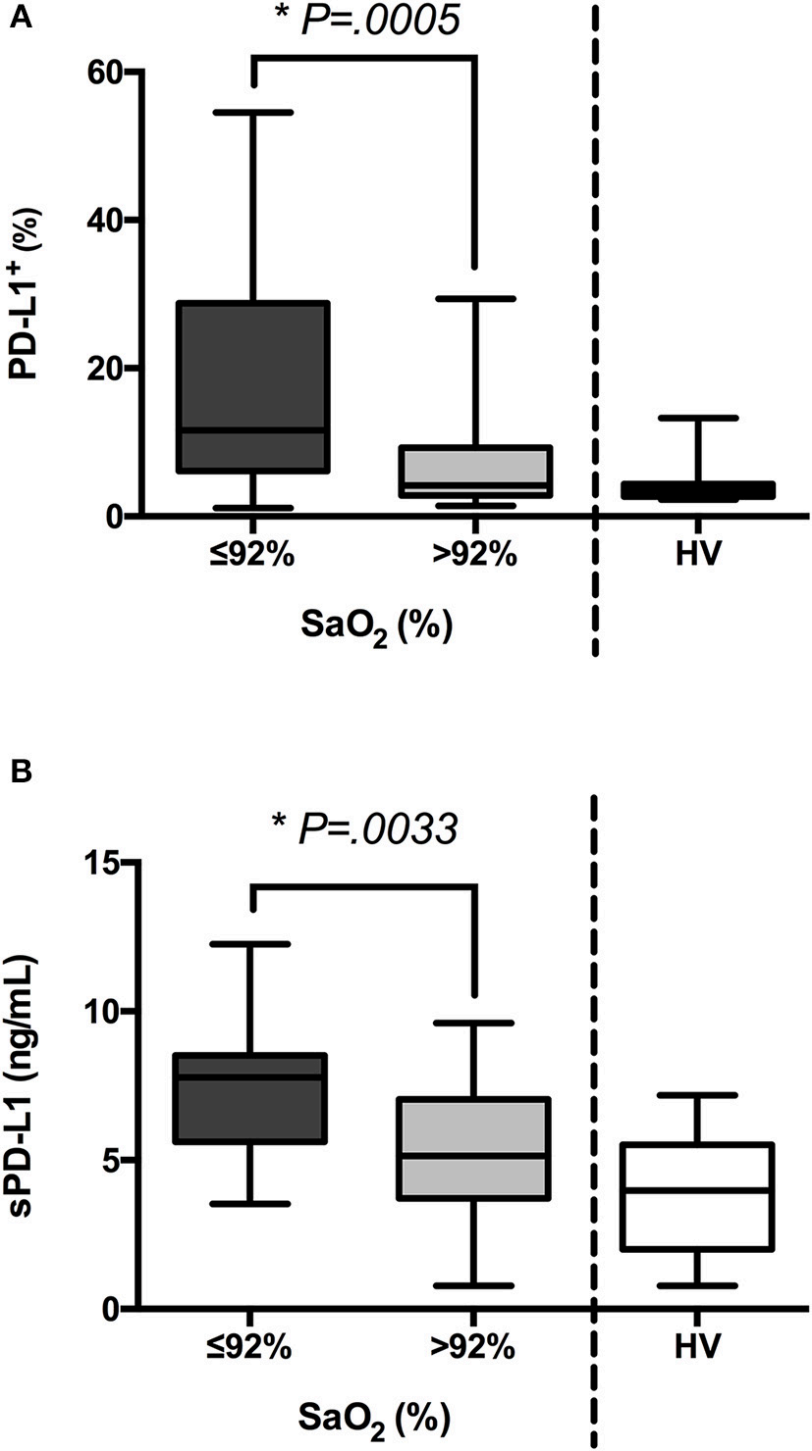
PD-L1 and sPD-L1 levels are increased in patients with low oxygen saturation. **(A)** Blood samples from patients with sepsis (*n* = 85) and healthy volunteers (HV, *n* = 15) were stained with anti-PD-L1 antibody. Next, percentages of PD-L1^+^ cells were analyzed on the gate of CD14^+^ cells by FACS. Percentages of CD14^+^PD-L1^+^ cells in patients classified according to their oxygen saturation are shown. **(B)** Concentrations of sPD-L1 were quantified in sera from septic patients and HV by ELISA. ^*^p < 0.05 using a Student's *t*-test.

**Figure 5:**
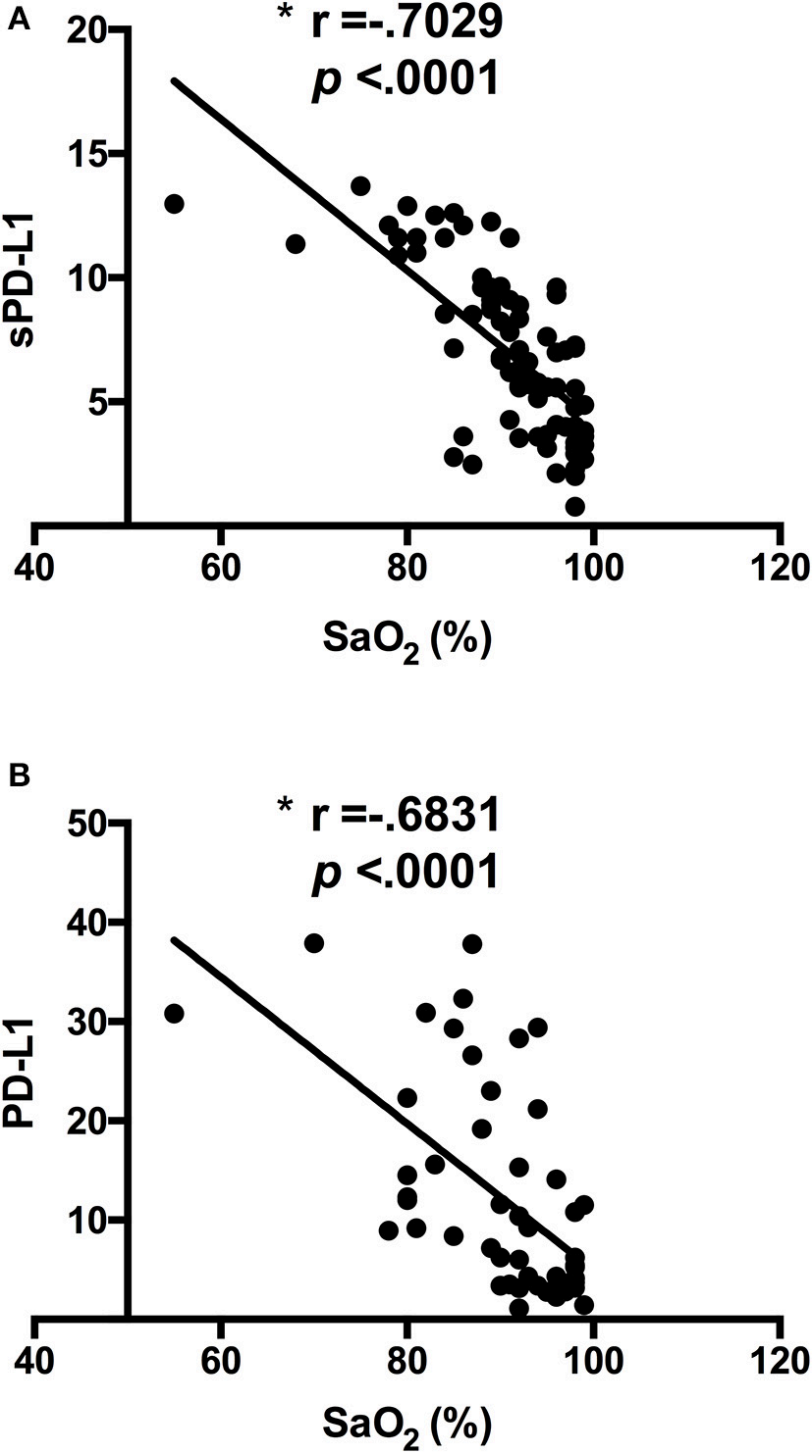
Levels of both sPD-L1 in sera and PD-L1 on CD14^+^ cells correlate with oxygen saturation in patients with sepsis. **(A)** Concentrations of sPD-L1 were quantified in sera from patients with sepsis (*n* = 85) by ELISA. The correlation between sPD-L1 and levels of oxygen saturation is shown. **(B)** Percentages of PD-L1^+^ cells gated on CD14^+^ cells from patients with sepsis (*n* = 62) were analyzed by FACS. The correlation between PD-L1 and levels of oxygen saturation is shown. ^*^p < 0.05 using Spearman's test.

Previously, we have reported the expression of HIF1α in circulating monocytes during sepsis (13, 15). In addition, we have learned that HIF1α governs the expression of the immune checkpoint ligand PD-L1 on monocytes, a crucial factor in T cell exhaustion induction (13, 15). Here, we observed a significant increment in HIF1α transcription in patients from group I (Supplementary Figure 2). In line, low levels of SaO_2_ linked to both high PD-L1 expression on circulating monocytes and an elevated concentration of sPD-L1 in sera (Figure 4), and there were inverse correlations between SaO_2_/sPD-L1 and SaO_2_/PD-L1 (Figure 5). These data and the results from the logistic regression model (Table 1) indicated a patent association between oxygen saturation and PD-L1 expression.

**Figure 6:**
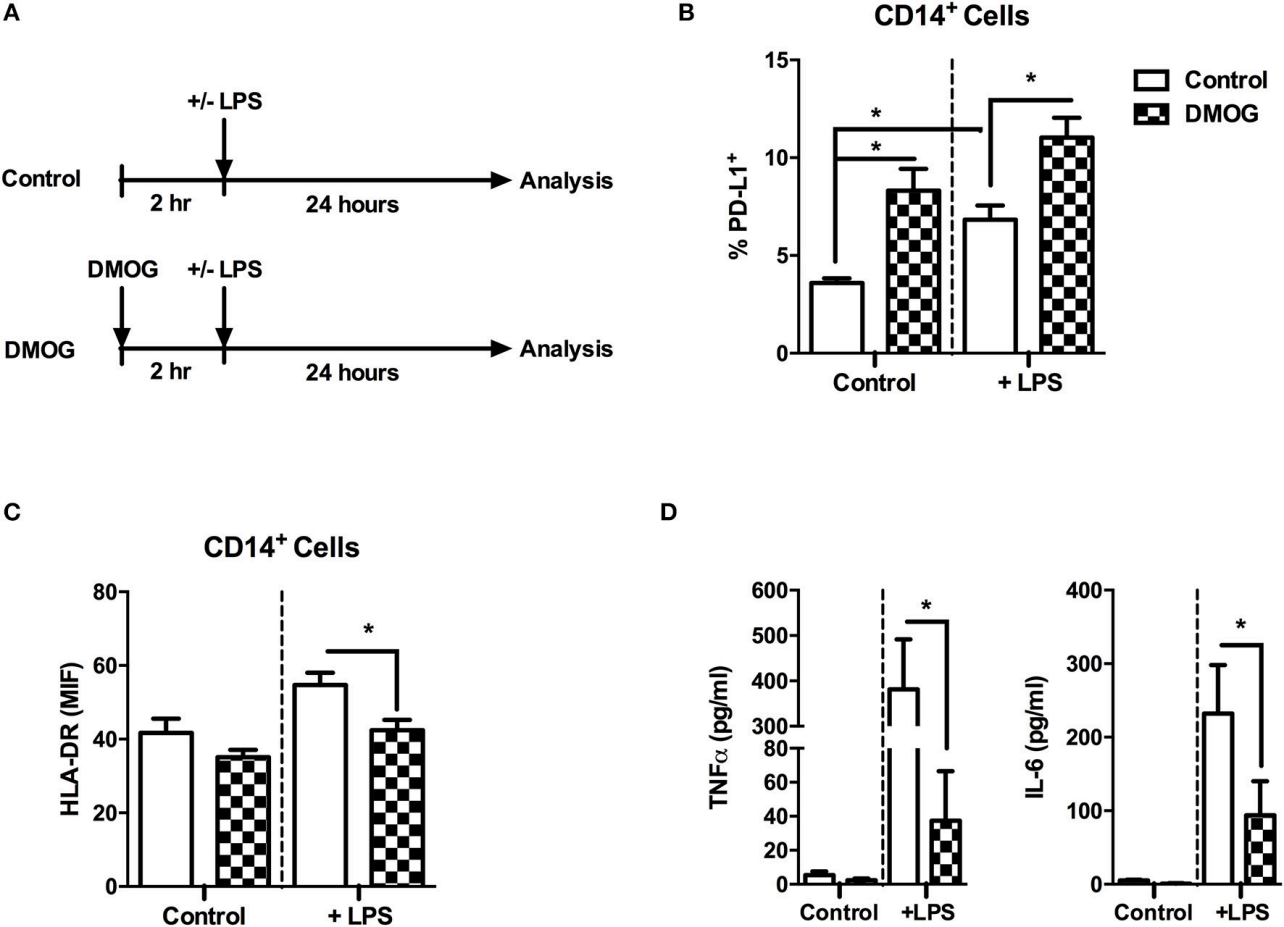
PHDs inhibition with DMOG causes PD-L1 overexpression and impaired LPS response on healthy monocytes. Monocytes from HV were isolated by ficoll and plate adherence, and stimulated following the represented scheme **(A)**. **(B)** Percentages of PD-L1^+^ cells gated on CD14^+^ cells. **(C)** Mean intensity fluorescence of HLA-DR on gated CD14^+^ cells. **(D)** TNFα and IL-6 production (pg/mL) after 24 h of *in vitro* LPS stimulation (10 ng/mL). ^*^p < 0.05 using a Student's *t*-test.

Since during hypoxemia the inhibition of prolyl hydroxylases (PHD) takes place, we examined the role of these enzymes in an *in vitro* model based on dimethyloxallyl glycine (DMOG)-treatment before LPS stimulation (Figure 6A). Treatment of peripheral blood human monocytes, isolated from healthy donors, with DMOG showed PD-L1 overexpression compared to the untreated cells reaching levels slightly superior to those raised when they were stimulated with LPS (Figure 6B). Furthermore, treatment with DMOG previous to LPS stimulation reduced both HLA-DR expression and cytokine production (Figures 6C,D, respectively). Thus, PHD inhibition reproduces some of the immunological features observed on hypoxemic septic patients suggesting its role in the control of the innate immune response during infections.

### PD-L1/PD-1 crosstalk blocking restores the immune response in patients with low oxygen saturation

**Figure 7:**
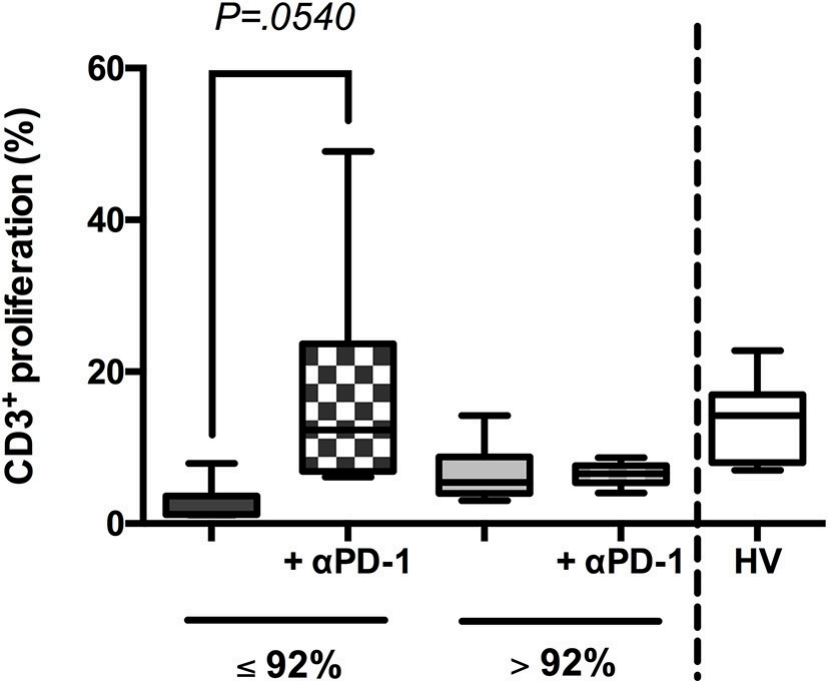
Blocking PD-L1/PD-1 crosstalk restored immune response in patients with low oxygen saturation. Peripheral blood mononuclear cells (PBMCs) were isolated from 25 randomly selected patients with sepsis (SaO_2_ ≤ 92%, *n* = 13 and SaO_2_ > 92%, *n* = 12) and HV (*n* = 5), labeled with CFSE and stimulated or not with PWD (2.5 μg/mL) for 5 days, in presence or not of an anti-PD-1 [αPD-1, (13)]. Then, proliferation of CD3^+^ cells was analyzed by FACS. Percentages of proliferation are shown in patients classified according to their oxygen saturation and HV. *P*-value using two-tailed Student's *t*-test.

Eventually, to study the PD-L1/PD-1 crosstalk implication in the observed impaired immune response in patients with low SaO_2_, a blocking assay using a commercial anti-PD-1 antibody was performed. Standard levels of CD3^+^ cell proliferation were observed in the presence of anti-PD-1 antibody, indicating a patent immune response recovery (Figure 7).

## Discussion

Although the harmful features of sepsis are thought to be due to the damaging effects caused by over inflammation (20, 26), a number of anti-inflammatory therapies, including anti-endotoxin (27), anti-TNFα (28, 29), anti-IL1 (30), and Toll-like receptor inhibitors (31, 32), have failed in some clinical trial phases. In contrast, recent data highlight the relevance of immunosuppression (6, 33, 34) and the alternative non-inflammatory activation (15, 25) of the innate immune system in sepsis evolution. Death because of sepsis, in most cases, is not due to over inflammation, which can be controlled with antibiotics and steroids, but instead reflects host immunosuppression, which confers a high risk of reinfection (6, 35).

Several studies on patients with sepsis have reported the upregulation of PD-L1^+^ monocytes (10, 11, 14). Two observational studies have been developed to identify potential changes in the PD-L1/PD-1 crosstalk during sepsis (NCT01161745 and NCT01976884). However, patients with sepsis showed a wide range of PD-L1 expression, and the potential benefit of this immunotherapy would be linked to the levels of this IC. In line, we have showed that in a cohort of septic patients only one third of them could benefit from an anti-PD-1 therapy (13).

In the current study, we identified on admission those patients who expressed high levels of both PD-L1 and sPD-L1. According to the data presented here, levels of oxygen saturation classified patients with sepsis into two groups that showed statistically different levels of PD-L1 and sPD-L1. In addition, those patients under 92% SaO_2_ (group I) exhibited not only an impaired inflammatory response, reduced antigen presentation and diminished adaptive response but also a poor prognosis with higher frequency of reinfection and mortality than those patients over 92% SaO_2_ (group II).

The analysis of HIF1α mRNA also revealed increased expression of this transcription factor in the group I of patients. We have previously described a crucial role for HIF1α in the control of hallmarks of sepsis evolution such as downregulation of proinflammatory cytokine production, PD-L1 expression on circulating monocytes, and subsequently, impaired T cell proliferation or adaptive response in the reinfection context (13, 15). Moreover, our *in vitro* data indicated that prolyl hydroxylases inhibition by DMOG reproduced the hallmarks of hypoxemic septic patients. In this regard, the correlation between SaO_2_ and PD-L1 expression provides a useful tool for stratification of patients with sepsis on admission, indicating those candidates suited for anti-PD-1 immunotherapy, which would prevent a failure of the immune response.

Recently, blockage of PD-L1/PD-1 crosstalk using an anti-PD1 antibody has meant a revolutionary treatment for many types of tumors, such as melanoma, lung, and renal cancers (9, 36, 37). In a mouse model of sepsis, administration of anti-PD-L1/PD-1 antibodies prevented lymphocyte depletion (38) and improved survival (39), suggesting the need for its translational implementation in human patients. However, not all septic patients can benefit from this therapy. Apparently, only those who show expression of PD-L1 on monocytes and its receptor on lymphocytes will be prone to it. Here we report that SaO_2_, an easy-to-measure parameter, provides useful information about the expression of PD-L1 on monocytes from septic patients. Moreover, our results indicate that immunotherapy with anti-PD-1 improves the adaptive response in those patients with low SaO_2_ (≤ 92%, group I). Note that patients from this group also generated high severity scores (APACHE II and qSOFA) and expressed high levels of PD-L1 in their circulating monocytes, as well as showed elevated concentration of sPD-L1 in sera; hence, the lowest rate of T cell proliferation was corrected by blocking PD-L1/PD-1 crosstalk, as referenced before.

## Conclusions

The present study proposes SaO_2_ as a useful marker not only to predict sepsis evolution but also to identify potential immune-exhausted patients susceptible to personalized immunotherapy. Incorporating this indicator into routine emergency protocols for patients with sepsis might enhance their outcome.

## Author contributions

JA-O collected, analyzed, and interpreted the data. CM-E, CC-C, and AM-Q, recruited the patients and provided clinical data. VT, KM-H, EL-G, and RL-R processed blood samples and data. EL-G, PG-C, and LA supervised statistical analysis and revised the manuscript. EL-C devised and designed the study, interpreted the data and wrote the manuscript. All authors read and approved the final manuscript.

### Conflict of interest statement

The authors declare that the research was conducted in the absence of any commercial or financial relationships that could be construed as a potential conflict of interest.
