# Supplementary material for: Oxygen Saturation on Admission Is a Predictive Biomarker for PD-L1 Expression on Circulating Monocytes and Impaired Immune Response in Patients With Sepsis
Source: Front Immunol. 2018 Sep 4;9:2008. doi: 10.3389/fimmu.2018.02008 (PMC6131191; doi:10.3389/fimmu.2018.02008)
Supplement: Supplementary file 1 [file Image_1.pdf]

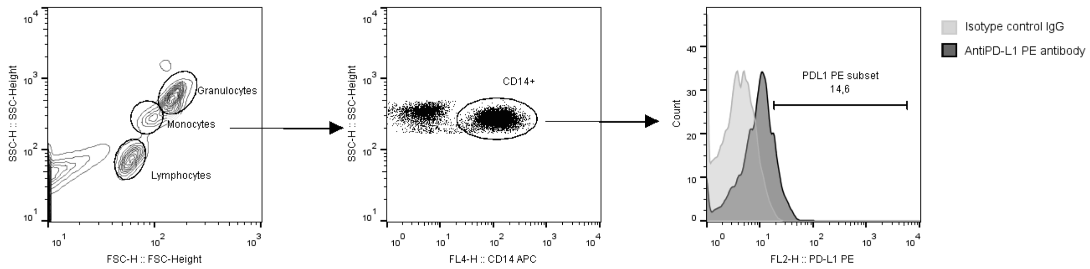

Supplementary Figure 1. Gating strategy to study PD-L1 expression on monocytes isolated from patients with sepsis.

Whole blood from patients were treated with BD FACS™ lysing solution (BD Biosciences) for lysing red blood cells according to manufacturers' instructions. Then, cells were staining with CD14-APC and PD-L1-PE antibodies or isotype control IgG for negative controls. Data were acquired in FACSCalibur flow cytometer (BD Biosciences) and analysed with FlowJo vX.0.7 software (FlowJo, LLC).
