# Supplementary material for: Oxygen Saturation on Admission Is a Predictive Biomarker for PD-L1 Expression on Circulating Monocytes and Impaired Immune Response in Patients With Sepsis
Source: Front Immunol. 2018 Sep 4;9:2008. doi: 10.3389/fimmu.2018.02008 (PMC6131191; doi:10.3389/fimmu.2018.02008)
Supplement: Supplementary file 2 [file Image_2.pdf]

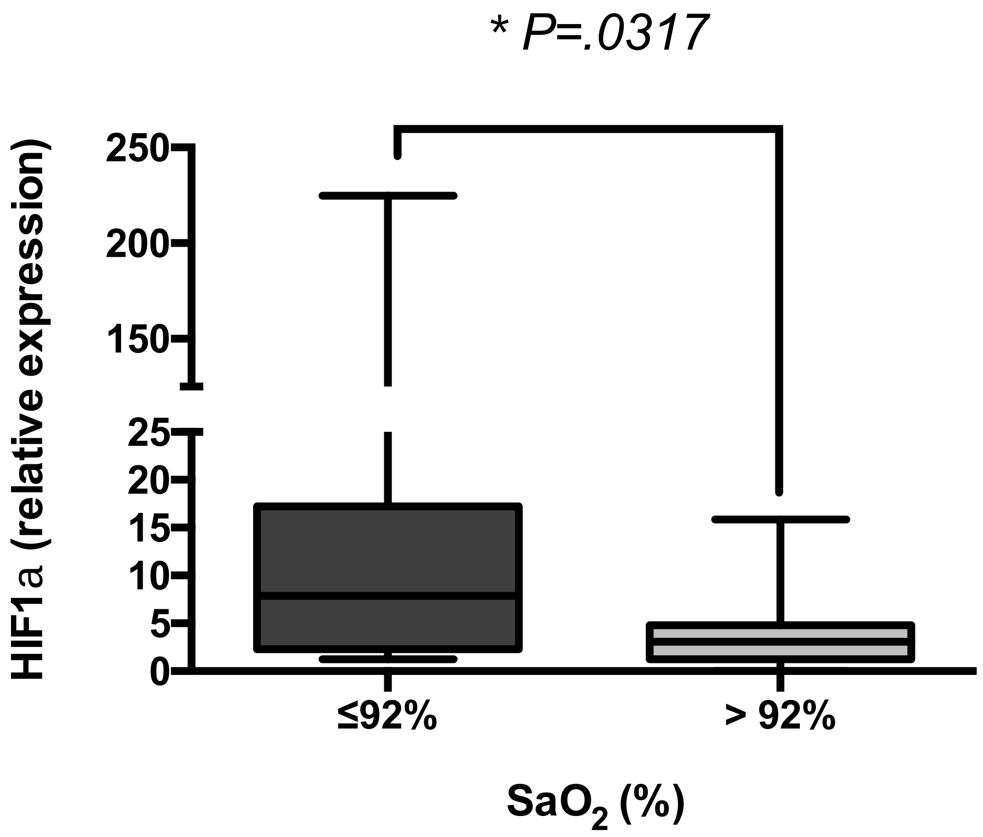

Supplementary Figure 2. HIF1α expression is higher in patients with low oxygen saturation.

Peripheral blood mononuclear cells (PBMCs) from patients with sepsis (n=40, randomly selected) were cultured on adherent plates for 1 hour. Then, adherent cells (≈90% $CD14^+$) were harvested and RNA isolated. Levels of HIF1α relative expression were analysed by real time Q-PCR. *, p<.05 using a Student's t-test.
